# Resource Utilization of Red Mud from the Solid Waste of Aluminum Industry Used in Geothermal Wells

**DOI:** 10.3390/ma15238446

**Published:** 2022-11-27

**Authors:** Zhiqiang Wu, Lihua Li, Fei Gao, Gaoyin Zhang, Jingxuan Cai, Xiaowei Cheng

**Affiliations:** 1 CNOOC Research Institute Co., Ltd., Beijing 100028, China; 2 CNPC Bohai Drilling Engineering Co., Ltd., Tianjin 300457, China; 3 School of New Energy and Materials, Southwest Petroleum University, Chengdu 610500, China

**Keywords:** geothermal, high belite cement, response surface method, industrial solid waste

## Abstract

It is difficult for the commonly used Class G oil well cement to withstand the high-temperature environment of geothermal wells, and it is easy to deteriorate the mechanical properties and damage the integrity of the cement sheath. Industrial solid waste red mud can be used as supplementary cementing materials (SCMs) to improve its mechanical properties at high temperatures. In addition, compared to Class G oil well cement, high belite cement (HBC) has lower energy consumption and better mechanical properties at high temperatures. In this study, the mechanical properties of HBC as a gel material and quartz sand and red mud as SCMs were studied at high temperatures. The ratio of HBC to SCMs and the ratio of quartz sand to red mud in SCMs were optimized using the response surface method (RSM). The response surface was established using the three-level factorial design model, which fit well with the experimental data. The optimization results show that the best mass ratio of SCMs/HBC is 37.5% and that the best quality ratio of quartz sand/red mud is 9 under the curing conditions of 180 °C. However, the best mass ratio of SCMs/HBC is 49.3%, and the best quality ratio of quartz sand/red mud is 7 under 220 °C. With the addition of SCMs, the silicon-to-calcium ratio of HBC hydration products decreases, and high-temperature-stable xonotlite and tobermorite can be formed. After adding SCMs, the cement sample is denser without obvious cracks.

## 1. Introduction

As a promising alternative to traditional fossil energy, geothermal energy plays an important role in providing thermal energy for humans. The extraction of geothermal energy is an important measure to achieve “carbon neutrality” [1]. Geothermal well power generation is the most promising way to use geothermal resources at high temperatures (above 150 °C). At present, China’s geothermal energy extraction is dominated by geothermal resources of about 180–220 °C [2]. According to the characteristics of geothermal resources, the main method is to produce hot water or steam to generate electricity. In order to ensure the mining performance of geothermal wells, the mechanical properties of the cementitious materials at high temperatures should be satisfied [3].

High-alite Class G cement is currently widely used as the cementitious material for oil wells. Alite cement, as a basic cementitious material, consumes a large amount of energy and emits a large amount of greenhouse gases during manufacturing. Even if the cement industry only accounts for approximately 7.4% of total CO_2_ emissions [4], it still has a large influence. As a kind of Portland cement, high belite cement (HBC) takes belite as the dominant mineral and increases its content to more than 30%. The advantage of HBC is that the low energy consumption and low-emission component, C_2_S, in the clinker replaces the high energy consumption and high-emission C_3_S as the main mineral component, and the calcination temperature of 1250 °C is also lower than the 1450 °C required for alite cement [5]. Compared to Portland cement, HBC can reduce energy by 16%, and the total CO_2_ emissions during cement production can be reduced by 10% [6]. HBC has advantages over alite cement due to its long-term strength and durability and is often used in dams and roads [7,8]. The shortcoming of HBC is its low early strength, but a high-temperature service environment can change this status [7,9].

For Portland cement, unstable hydration products are formed under high-temperature hydrothermal conditions. When the temperature exceeds 110 °C, the C-S-H gel phase initially formed by Portland cement hydration is transformed into hydrated dicalcium silicate crystals (α-C_2_SH) [10]. When the temperature rises further, α-C_2_SH transforms into jaffeite (C_6_S_2_H_3_) [11]. These two crystals have low strength and high permeability, and the increase in density causes the cement to shrink and stay. Larger pores will eventually lead to a decrease in compressive strength and an increase in permeability. Therefore, it is necessary to add some supplementary cementitious materials (SCMs) to Portland cement to improve its thermal stability.

SCMs often have a similar chemical composition to Portland cement and have gelling activity. In recent years, considering environmental protection factors, an increasing number of researchers have added waste materials to Portland cement as SCMs, such as slag, fly ash, and red mud [12,13,14], which can appropriately decrease costs while reducing environmental pollution and land resource occupation by these solid waste materials [15].

Red mud is the tailing produced during the production of alumina from bauxite [16]. Its appearance is due to its hematite content, which varies and appears reddish brown or grey [17,18]. At present, the main red mud treatment methods used in the world are the ocean discharge method, the lagoon method, and the dry accumulation method. The world accumulative reserves of red mud have exceeded 4 billion tons, but due to the very limited use of red mud in the industry, stock is constantly increasing, and the utilization rate is extremely low [19] The greatest impact on the environment from the disposal and storage of red mud is the contamination of soil and water bodies by residual liquids and suspensions [20,21].

Red mud contains SiO_2_, A1_2_O_3_, Fe_2_O_3_, CaO, and silicate minerals necessary for the mass production of cement, as well as a certain amount of amorphous aluminosilicate substances, which can react with water to hydrate [22,23,24,25]. In addition, the aluminosilicate in red mud undergoes a pozzolanic reaction under the action of Ca(OH)_2_ released during cement hydration and is a potentially active cementitious material [15]. Because of its high calcium content, Bayer-process red mud can be used as a raw material for cement production and can also be used as SCMs in cement [26,27,28].

Therefore, this study used HBC as a cementitious material and quartz sand and Bayer-process red mud as SCMs to explore the influence of SCMs on the strength of HBC at high temperatures and study the microscopic mechanism of the hydration reaction between SCMs and HBC. Based on the response surface method (RSM), the incorporation ratio of SCMs and the composition ratio of SCMs are optimized. By adding SCMs composed of quartz sand and red mud to HBC, it is possible to effectively decrease waste and reduce energy consumption while ensuring stable cement performance. This study provides a new way to solve the development of cementing slurry in geothermal wells, and also provides a certain experimental and theoretical basis. In addition, it provides a new development method to solve the problem of solid waste red mud piling up and realize the rational application of red mud resources.

## 2. Materials and Methods

### 2.1. Materials

Table 1 shows the mineral composition of HBC and red mud used in the experiment. The HBC came from Leshan Jiahua Special Cement Co., Ltd. The content of C_2_S in the HBC clinker increased to 36.97%, while the content of C_3_S decreased to 37.87%. The red mud was obtained from the Bayer process. The mineral composition of the red mud is listed in Table 2. From the table, it can be seen that the Bayer-process red mud contained cement-like components. Among them, the contents of silicon and aluminum were higher, the content of SiO_2_ reached 27.63%, the content of Al_2_O_3_ reached 26.44%, and the main component of the red mud was aluminosilicate. The quartz sand came from Sichuan Huaxi Group Co., Ltd., with a size of 150 mesh. The SiO_2_ content in the silica sand used in this experiment was greater than 96%.

The particle size of the HBC ranged from 0.18 to 126 μm, and the D50 was 12.23 μm. The particle size range of the red mud was 1~158 μm, and the D50 was 3.619 μm. The particle size range of the quartz sand was 0.89~158 μm, and the D50 was 46.407 μm. The particle size distribution of the material was measured by a laser particle size analyzer (Mastersizer 2000 laser particle size analyzer, Malvern Instrument Co., Ltd., Malvern, UK).

The red mud was dried using a GW300-PLC frequency conversion roller heating furnace (Qingdao Tongchun Petroleum Instrument Co., Ltd., Qingdao, China) at 105 °C for 2 days to remove most of the water, and then placed in a 60 °C electric air box (101-2 type, Beijing Zhongxingweiye Instrument Co., Ltd., Beijing, China) to remain dry.

The additives used in the experiment included a fluid loss agent (G33S) and dispersant (SXY-2). G33S is a polymer-modified material made of polyamide, low-molecular-weight polyamide, and hydroxypolycarboxylic acid, which is soluble in water. SXY-2 is a polymer formed by the coagulation of formaldehyde and acetone (Cheng et al., 2018).

**Figure 1:**
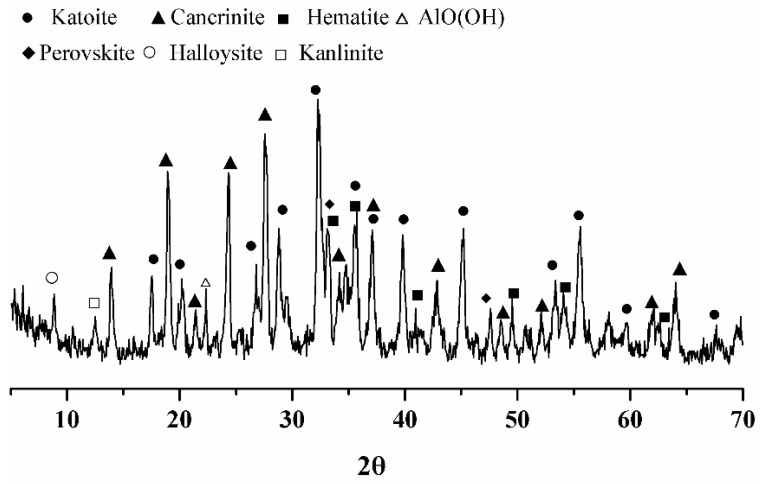
Phase composition of red mud.

The phase composition of Bayer red mud was obtained by X-ray diffraction analysis, as shown in Figure 1. It can be seen in the figure that the main mineral composition of the red mud was katoite (Ca_3_Al_2_(SiO_4_)(OH)_8_, C_3_A_S_H_4_, ICSD: 38-0368), cancrinite (Na_6_Ca_2_Al_6_Si_6_O_24_(CO_3_)_2_·2H_2_O, ICSD: 46-1332), hematite (Fe_2_O_3_, ICSD: 33-0664), perovskite (CaTiO_3_, ICSD: 22-0153), halloysite (Al_2_Si_2_O_5_(OH)_4_·2H_2_O, ICSD: 29-1489), kaolinite (Al_2_Si_2_O_5_(OH)_4_, ICSD: 14-0164), and AlO(OH) (ICSD: 05-0355) [12,29]. Most of the red mud was aluminosilicate material, of which cancrinite is an unstable material. Cancrinite and katoite were the main active mineral components in the red mud.

### 2.2. Methods

#### 2.2.1. Cement Slurry Preparation

The specific mix proportions of the cement slurry are shown in Table 3 and Table 4. The mixing procedure was conducted according to the API RP 10B-2 standard, in which a premixture was thoroughly and uniformly mixed with cement and dry additives.

#### 2.2.2. The Compressive Strength Test

Compressive strength tests were performed on the samples that had been cured in a high-temperature and high-pressure curing kettle at 110, 150, 180, and 220 °C for 7 days. According to the API RP 10B-2 standard, the specimens used for the compressive strength tests were cubes with 50-mm sides, and the tests were conducted with an electronically controlled hydraulic testing machine (TYE-300B, Wuxi Jianyi Instrument & Machinery Co., Ltd., Wuxi, China) operated with a load rate of 1200 ± 100 N/s.

#### 2.2.3. Characterization

X-ray diffraction (XRD) with Cu Kα radiation (DX-1000, V = 30 kV, I = 25 mA) was used to collect the sample data. The measurements were performed with a step size of 0.02° and a scan rate of 2°/min in the scan range of 5–70° 2 theta (2θ).

The weight loss of the samples after drying was measured by thermogravimetric analysis (TGA/SDTA85/e, METTLER TOLEDO, Greifensee, Switzerland). The temperature ranged from 105 to 800 °C. The heating rate was 10 °C/min. The protective gas was nitrogen with a flow rate of 50 mL/min.

Environmental scanning electron microscopy (ESEM) (Model Quanta 450, FEI, Morristown, NJ, USA) was used to assess the morphology of the samples. To minimize electric charging during the measurement, the samples were sputtered with a fine layer of gold.

The pore size distribution of the sample was measured by a specific surface area and pore size distribution instrument (F-Sorb3400, Beijing Jin Aipu Technology Co., Ltd., Beijing, China).

## 3. Results and Discussion

### 3.1. Single-Factor Tests

#### 3.1.1. Influence of the Supplementary Cementitious Material/Cement (SCM/Cement) Mass Ratio on Compressive Strength

**Figure 2:**
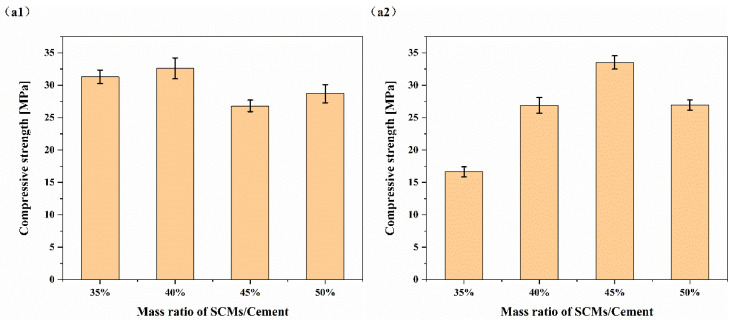
Influence of the SCM/cement mass ratio on compressive strength: (**a1**) 180 °C; (**a2**) 220 °C.

The mass ratio of quartz sand/red mud (Q/R) was maintained at 4, and the influence of the SCM/cement mass ratio on the compressive strength is shown in Figure 2. SCMs can improve the compressive strength of cement in high-temperature environments, and they have similar effects on the improvement of the high-temperature compressive strength of HBC. Under curing at 180 °C, when the content of the SCMs was 35%, the compressive strength was 31.30 ± 1.04 MPa, and when the content increased to 40%, the compressive strength increased slightly to 32.61 ± 1.62 MPa. With the increase in the content again, the compressive strength decreased; the compressive strength of cement was not sensitive to the amount of SCMs added. However, under curing at 220 °C, the compressive strength of the cement further declined. With the increase in SCMs, the compressive strength of the cement could be improved. With the increase in content, the compressive strength of the cement paste showed a trend of increasing and then decreasing. When the dosage was 45%, the compressive strength improvement effect was at its peak. The compressive strength was 33.51 ± 1.03 MPa. It is worth noting that when the SCM content was 45%, the compressive strength of cement stone cured at 220 °C was higher than that at 180 °C. This may have been due to the formation of a dense aggregate structure of the hydration products at high temperatures; the pores in the cement paste were reduced, the thermal conductivity was increased, and then the internal thermal stress of the cement paste was reduced. The resulting thermal stress damage to the cement stone was reduced.

#### 3.1.2. Influence of the Q/R Mass Ratio on Compressive Strength

**Figure 3:**
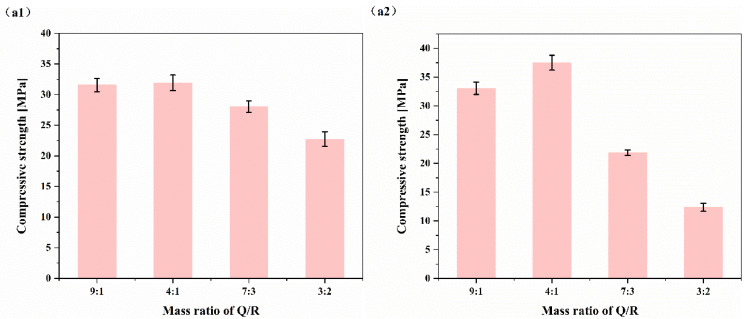
Influence of the Q/R mass ratio on HBC compressive strength: (**a1**) 180 °C; (**a2**) 220 °C.

The mass ratio of SCMs/cement was maintained at 45%, and the influence of the Q/R mass ratio on the compressive strength is shown in Figure 3. For HBC, the effect of improving the compressive strength of cement was best when the mass ratio of Q/R was 4. At this time, the compressive strength of the cement paste cured at 180 °C reached 31.92 ± 1.27 MPa, while it was 37.50 ± 1.31 MPa at 220 °C. As the amount of red mud increased, the compressive strength declined significantly. This was because the part of the red mud that could participate in the reaction is limited at high temperatures, and the incorporation of too much red mud can only have a filling effect on the cement slurry and occupy the space that originally belongs to the hydration product, so the strength of the cement paste decreases.

### 3.2. RSM Experiment

#### 3.2.1. Experimental Design

Based on the single-factor experimental results, the compressive strength was the highest when the Q/R mass ratio was 4. With the difference in the curing temperature, the increase in the mass ratio of SCMs had different effects on the improvement of the cement’s compressive strength. Thus, under curing temperatures of 180 and 220 °C, the raw material ratios were set close to these values in the RSM design. In this study, the three-level factorial design model in the RSM was adopted, and compressive strength was chosen as the response value.

Experiments of two factors and three levels were designed. The two factors were the mass ratios of the SCMs/cement and Q/R, and the three levels were set as −1, 0, and 1. The experimental design was carried out using Design Expert software (version 12). The settings of the factors and levels are shown in Table 5.

According to the design in Table 4, cement samples were prepared with HBC and tested for compressive strength after curing at 180 and 220 °C for 7 days.

#### 3.2.2. Establishment and Significance Test of the RSM Model

##### Cement Samples Prepared with HBC

Polynomial fitting was performed on the data in Table 6 and Table 7. The regression equation obtained was expressed as:

(1) $$Y_{180{}^{\circ}C}=31.97-0.945A+5.13B-1.81AB-3.59A^{2}-2.09B^{2}-0.8575A^{2}B-2.52AB^{2}\;\left(R_{Adj}^{2}=0.9221\right)$$

(2) $$Y_{220{}^{\circ}C}=24.77+10A+10.13B-2.02AB+1.31A^{2}-1.69B^{2}-5.14A^{2}B-6.17AB^{2}\;\left(R_{Adj}^{2}=0.9807\right)$$

where *A* and *B* represent the mass ratios of SCMs/cement and Q/R, respectively, and *Y* represents the compressive strength as the response value.

The analysis results of the regression model are shown in Table 8. As shown in Table 8, after curing at 180 °C, the Model F-value of 6.76 implies that the model is significant. There is only a 4.19% chance that this large F-value could occur due to noise. The lack of fit F-value of 7.28 (*p*-value > 0.05) implies that the lack of fit is not significant relative to the pure error. Therefore, the model fit the data well and was applied to describe the relationship between the mass ratios of the raw materials and the compressive strength of the cement after curing at 180 °C.

As shown in Table 9, after curing at 220 °C, the model *p*-value of 0.0199 implies that the model is significant. Owing to the noise, the occurrence probability of this F-value was only 0.28%. The lack of fit *p*-value of 0.0906 implies that the lack of fit is not significant relative to the pure error. Therefore, the model fit the data well and was applied to describe the relationship between the mass ratios of the raw materials and the compressive strength of the cement after curing at 220 °C.

#### 3.2.3. Optimization and Verification

**Figure 4:**
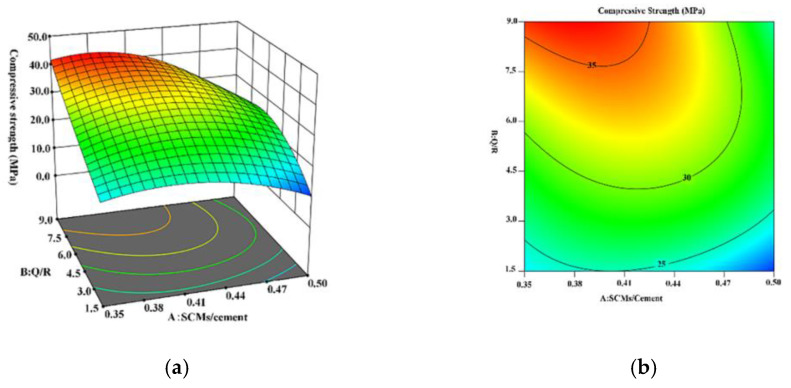
Diagram of the RSM model of HBC after curing at 180 °C: (**a**) 3D response surface; (**b**) contour line diagram of the model.

**Figure 5:**
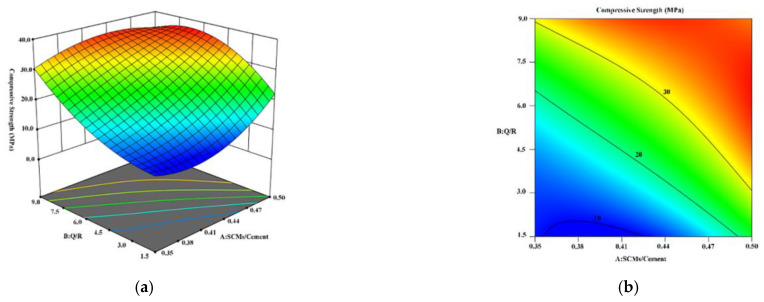
Diagram of the RSM model of HBC after curing at 220 °C: (**a**) 3D response surface; (**b**) contour line diagram of the model.

According to the regression Equations (1) and (2) and test data obtained via the RSM experiment, the 3D response surface and contour diagrams were drawn to exhibit the relationship between the compressive strength of the HBC and the mass ratios of the SCMs, as presented in Figure 4 and Figure 5. Figure 4 shows that the extreme values of the compressive strength of the sample after curing at 180 °C appeared in the red region at the SCM/cement mass ratios of approximately 0.38–0.41 and Q/R mass ratios of 8.5–9.0. The highest compressive strength was 37.09 MPa when the mass ratios of the SCMs/cement and Q/R were 0.375 and 9, respectively.

Figure 5 shows the result of the RSM model of HBC after curing at 220 °C. Large fluctuations in the compressive strength can be seen. The distribution position of the blue area shows that, when the mass ratio of the SCMs/cement was lower than 4, the compressive strength of the cement had a cliff-like drop. According to the distribution position of the red area, the highest compressive strength was 38.51 MPa when the mass ratios of the SCMs/cement and Q/R were 0.493 and 7, respectively.

### 3.3. Phase Analysis of Hydration Products

**Figure 6:**
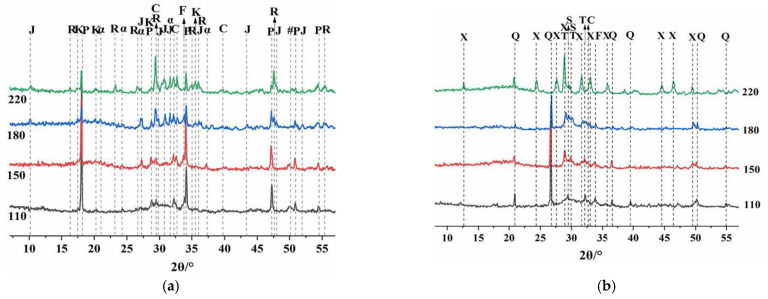
XRD patterns of cement samples in a high-temperature environment (α = α-C_2_SH; C-C_2_S; K—Katoite; P—Portlandite; J—Jaffeite; F—Ferrite; # = Ca_1.5_SiO_3.5_·xH_2_O; Q-Quartz; X—Xonotlite; T—Tobermorite; S—Scawtite; R—Reinhard bauxite). (**a**) HBC. (**b**) HBC with quartz sand.

Figure 6 shows the phase composition and changes in the cement sample under a high-temperature environment. To explore the mechanism of the effect of SCMs on the mechanical properties of HBC at high temperatures, X-ray diffraction analysis was performed on cements of different compositions. The low compressive strength of cement at high temperatures is due to the formation of C_6_S_2_H_3_ by a series of dissolution and precipitation processes, such as the aggregation of Ca^2+^ of C-S-H, which causes higher permeability in cement [11,30,31]. Before adding the quartz sand, CH was the dominant phase in the HBC. As the temperature rose, α-C_2_SH, C_6_S_2_H_3_, and Reinhard bauxite (C_5_S_2_H) appeared. After the quartz sand was added, diffraction peaks of the silicon phase (quartz, 2θ = 26.77°, 50.28°) appeared, and the peak intensity gradually weakened as the temperature rose, which indicates that, as the temperature rose, the amount of quartz sand consumed increased. Quartz sand can reduce the ratio of Ca/Si of hydration products and generate C_6_S_6_H (xonotlite, 2θ = 28.90°, 49.15°, etc.) [32,33] without generating hydration products with a high ratio of Ca/Si, such as C_6_S_2_H_3_. In addition, the presence of tobermorite (C_5_S_6_H_5_) was found in the hydration product phase at 150 °C and 180 °C. As the temperature increased, the production of C_6_S_6_H also increased. Meanwhile, scawtite (2θ = 29.47°, 29.99°), which is formed by the carbonization of C-S-H at high temperatures, was also found in the diffraction pattern. A small amount of scawtite is beneficial for increasing the strength of cement.

**Figure 7:**
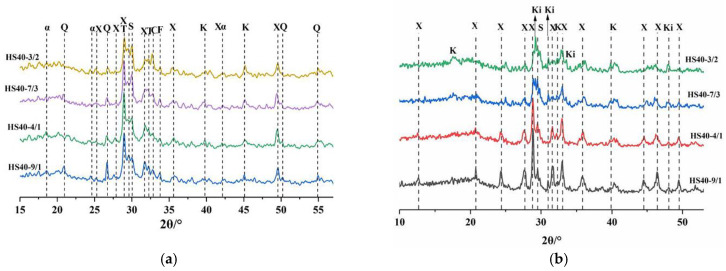
The effects of SCMs with different Q/R mass ratios on the XRD patterns of HBC hydration products at high temperatures (α = α-C_2_SH; C-C_2_S; K—Katoite; F—Ferrite; Q—Quartz; X—Xonotlite; T—Tobermorite; S—Scawtite; Ki—Kilchoanite). (**a**) 180 °C. (**b**) 220 °C.

Figure 7 shows the effects of SCMs with different Q/R mass ratios on the XRD patterns of HBC hydration products at high temperatures. As the amount of red mud substitution increased, the content of the silicon phase decreased, and the diffraction peak of this phase gradually weakened. Because the red mud contained a large amount of katoite, its relative diffraction peaks were enhanced from HS to HS40-3/2. In addition, the peaks after adding red mud were obviously more chaotic and disorderly. At 220 °C and a Q/R of 1.5, the presence of Ca_6_(SiO_4_) (Si_3_O_10_) (Kilchoanite, 2T = 32.92°) was also found.

**Figure 8:**
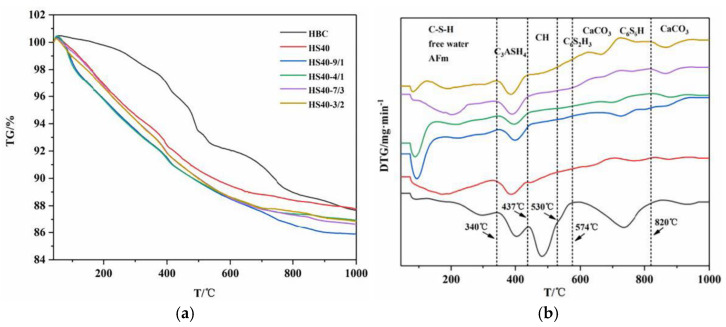

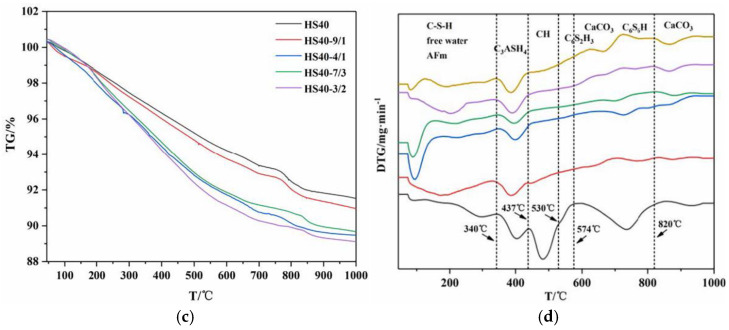
TG and DTG curves of the cement sample after curing at high temperature. (**a**) TG curves (180 °C). (**b**) DTG curves (180 °C). (**c**) TG curves (220 °C). (**d**) DTG curves (220 °C).

Figure 8 shows the thermal weight loss curve of the sample at high temperatures. It can be seen from the figure that the cement sample, after the addition of quartz sand, had no obvious weight loss peaks of CH and C_6_S_2_H_3_, but there were weight loss peaks of scawtite, xonotlite, and calcite in the range of 574~820 °C. The specific weight loss data of each temperature section are listed in Table 10. In the curve of the cement sample cured at 180 °C, the pyrolysis peak at 0–340 °C indicates the thermal decomposition of the C-S-H gel and a portion of the free water and bound water. After adding SCMs, the calcium-to-silicon ratio of the hydration product was reduced, which avoided the process of C-S-H conversion to C_6_S_2_H_3_ and increased the mass loss in this section. The pyrolysis peak at 340–437 °C indicates the increase in the hydrated garnet caused by red mud. The amount of pyrolysis at 437–574 °C was significantly reduced, which indicates that SCMs could consume Ca^2+^, thereby reducing the conversion of Ca^2+^ to CH. However, as the proportion of red mud in the SCMs increased, the silicon-phase content decreased, and CH was generated again. There was decomposition of the scawtite, xonotlite, and calcite in the range of 574~820 °C. The decomposition of calcium carbonate also occurred in the 820~1000 °C section. Due to the decrease in the CH content, the content of calcium carbonate also decreased.

In the curve of the cement sample cured at 220 °C, weight loss in the range of 630–735 °C can be clearly observed, which may have been due to the decomposition of scawtite. The weight loss peak at approximately 800 °C was mainly due to the decomposition of xonotlite.

### 3.4. Micromorphology and Pore Size Distribution

**Figure 9:**
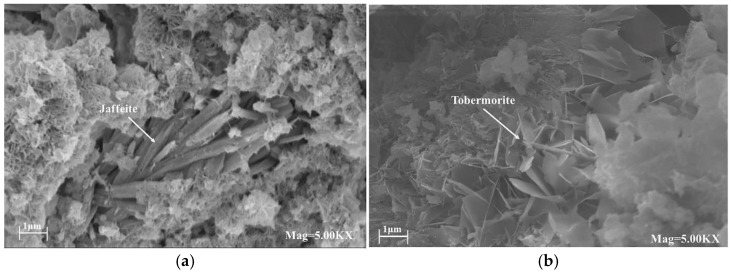

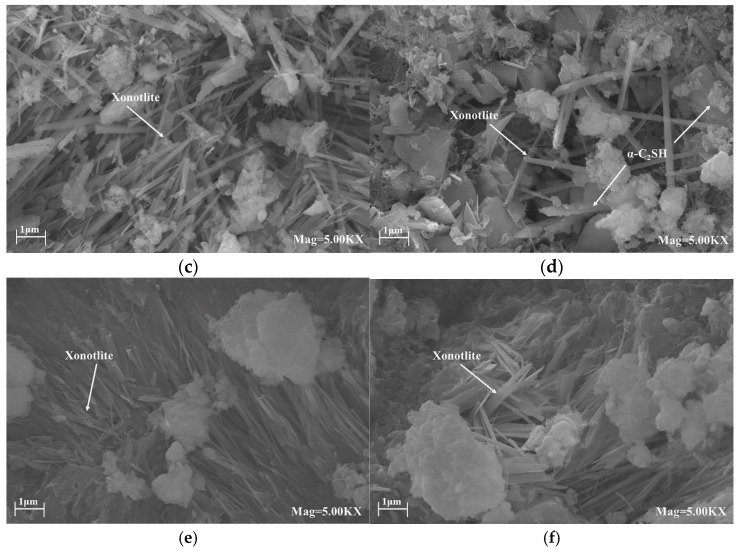
The micromorphology of cement samples. (**a**) HBC (180 °C). (**b**) HS40 (180 °C). (**c**) HS40-9/1 (180 °C). (**d**) HS40-3/2 (180 °C). (**e**) HS40-9/1 (220 °C). (**f**) HS40-3/2 (220 °C).

Figure 9a shows that, after curing at 180 °C, the HBC had large pores inside the cement due to the transformation of the hydrated product crystals. From the micromorphology of the cement sample, the high-temperature environment caused the surface of the cement to appear loose and shed small particles, and aggregated C_6_S_2_H_3_ crystals could be observed. Compared to the HBC, the micromorphology of the cement sample with quartz sand at 180 °C was denser, without obvious cracks. In addition, honeycomb-shaped tobermorite crystals can be observed in the figure, with Ca/Si = 0.83, which is a silicate material with high-temperature stability. Many acicular xonotlite crystals could be found in the micromorphology of the cement with a small amount of red mud. In contrast, no flaky, plate-like, or long fiber-like crystals were found, indicating that there was almost no CH or jaffeite. However, with the increase in the amount of red mud substitution, the densely arranged xonotlite in the staggered arrangement became dispersed, and at the same time, obvious plate-like crystals of α-C_2_SH could be observed.

Figure 9e,f shows the microscopic morphology of the cement sample at 220 °C. Figure 9e shows the microscopic morphology when the Q/R ratio was 9, and the denser xonotlite aggregation can be observed without obvious cracks and pores. Figure 9f shows the microscopic morphology when the Q/R ratio is 1.5. Compared to Figure 9e, the cement structure had decreased compactness and increased pores.

**Figure 10:**
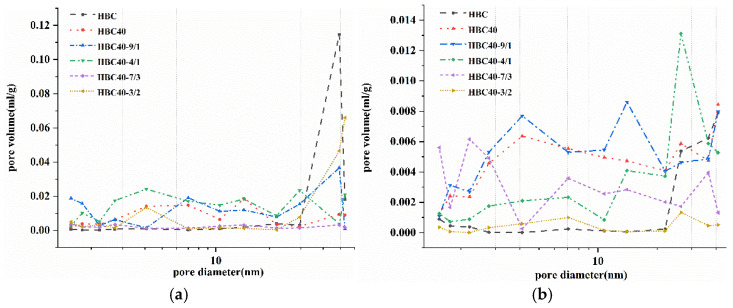
The pore size distribution of cement samples. (**a**) 180 °C. (**b**) 220 °C.

Figure 10 shows the pore size distribution within 100 nm of the different samples measured by the nitrogen adsorption method. The pores in the cement paste could be roughly divided into gel pores (<4.5 nm), mesopores or small capillary pores (4.5–5.0 nm), mesopores (50–100 nm), big wool stoma (100–1000 nm), and macropores (>10,000 nm). The results show that the number of gel pores of the HBC was very low. After adding the SCMs, the 0–20 nm pores inside the cement paste showed a rising trend, and the micropore volume above 20 nm showed a gradually decreasing trend. This shows that, during the HBC hydration process of the SCMs, the structure of the hydration product at high temperatures was transformed by changing the calcium-to-silicon ratio. This indicates that the SCMs significantly affected the pore structure of the cement paste. Due to its filling effect and participation in the hydration reaction process, the large pores in the cement paste were reduced and gradually developed into tiny pores. The macropores in the HBC hydration process were transformed into micropores with smaller pore diameters, which made the cement structure more compact. With the increase in the amount of red mud substitution, the number of pores decreased. This is in agreement with the previous results for compressive strength and microscopic morphology observations. When the Q/R ratio was 1.5, the 0–20 nm pore distribution inside the cement stone decreased, and the micropore volume above 20 nm had a gradually increasing trend.

## 4. Conclusions

(1) Using quartz sand and red mud as SCMs can improve the mechanical properties of HBC at high temperatures. Based on the RSM, under the curing condition of 180 °C, the best mass ratio of the SCMs/HBC was 37.5%, and the best quality ratio of the Q/R was 9. Under the curing condition of 220 °C, the best mass ratio of the SCMs/HBC was 49.3%, and the best quality ratio of the Q/R was 7.
(2) After adding SCMs, the cement system generated xonotlite and tobermorite at high temperatures. As the temperature increased, the amount of xonotlite produced also increased, and there was no CH. From HS to HS40-3/2, the relative diffraction peaks of katoite were enhanced.
(3) Based on the microscopic morphology and pore size distribution test results, the HBC sample was denser after adding SCMs without obvious cracks. No sheet-like CH, plate-like α-C_2_SH, or long-fibrous crystals of C_6_S_2_H_3_ were found. With the increase in the amount of red mud substitution, the densely packed xonotlite, which was originally staggered, became dispersed, and the dense space also formed pores. The number of pores within 100 nm of the cement cured at 220 °C was lower than that of the cement cured at 180 °C, indicating that the cement structure was denser at 220 °C.

## Figures and Tables

**Table 1:** Chemical and mineral composition of materials (wt.%).

Oxides	CaO	SiO_2_	Al_2_O_3_	Fe_2_O_3_	MgO	Na_2_O	TiO_2_	SO_3_	LOI	C_3_S	C_2_S	C_3_A	C_4_AF
HBC	62.42	22.88	4.26	4.71	1.52	0.41	—	2.43	0.56	37.87	36.97	3.3	14.32

**Table 2:** The oxide composition of red mud.

Title	Class			
	This Study	N	F	C
SiO_2_ plus Al_2_O_3_ plus Fe_2_O_3_, %	62.36	70.0	70.0	50.0
SO_3_, %	2.22	4.0	5.0	5.0
Moisture content, %	2.26	3.0	3.0	3.0
Loss on ignition, %	12.28	10.0	6.0	6.0

**Table 3:** The mix proportions of the cement slurry.

Group	HBC	SCMs	G33S	SXY-2	Water/Solid
HBC	100%	0%	1.5%	0.5%	44%
HS35	100%	35%	1.5%	0.5%	44%
HS40	100%	40%	1.5%	0.5%	44%
HS45	100%	45%	1.5%	0.5%	44%
HS50	100%	50%	1.5%	0.5%	44%

**Table 4:** The mix proportions of SCMs.

Group	SCMs	
	Quartz Sand	Red Mud
HS	100%	0%
HS-9/1	90%	10%
HS-4/1	80%	20%
HS-7/3	70%	30%
HS-3/2	60%	40%

**Table 5:** Factors and levels of RSM.

Factors Codes	Codes	Levels of Codes		
		−1	0	1
SCMs/Cement	A	0.35	0.425	0.5
Q/R	B	1.5	5.25	9

**Table 6:** Experimental results of cement samples prepared with HBC after curing at 180 °C.

No.	Levels of Codes		Experimental Values		Response Values
	A	B	SCMs/Cement	Q/R	Compressive Strength/MPa
1	0	−1	0.425	1.5	26.34
2	0	0	0.425	5.25	31.33
3	0	1	0.425	9	36.60
4	−1	1	0.35	9	35.03
5	0	0	0.425	5.25	30.12
6	1	1	0.5	9	24.49
7	1	−1	0.5	1.5	19.56
8	−1	−1	0.35	1.5	22.87
9	1	0	0.5	5.25	29.03
10	0	0	0.425	5.25	30.07
11	−1	0	0.35	5.25	30.92
12	0	0	0.425	5.25	33.16

**Table 7:** Experimental results of cement samples prepared with HBC after curing at 220 °C.

No.	Levels of Codes		Experimental Values		Response Values
	A	B	SCMs/Cement	Q/R	Compressive Strength/MPa
1	0	−1	0.425	1.5	11.34
2	0	0	0.425	5.25	24.33
3	0	1	0.425	9	37.60
4	−1	1	0.35	9	29.87
5	0	0	0.425	5.25	25.92
6	1	1	0.5	9	33.49
7	1	−1	0.5	1.5	21.56
8	−1	−1	0.35	1.5	9.84
9	1	0	0.5	5.25	37.47
10	0	0	0.425	5.25	22.87
11	−1	0	0.35	5.25	17.47
12	0	0	0.425	5.25	23.16

**Table 8:** Analysis of the variance of the regression model (after curing at 180 °C).

Source	Sum of Squares	df	Mean Square	F-Value	*p*-Value	Note
**Model**	255.28	7	36.47	6.76	0.0419	significant
A-SCMs/Cement	1.79	1	1.79	0.3311	0.5958	-
B-Q/R	52.63	1	52.63	9.76	0.0354	-
AB	13.07	1	13.07	2.42	0.1946	-
A^2^	34.34	1	34.34	6.37	0.0651	-
B^2^	11.69	1	11.69	2.17	0.2150	-
A^2^B	0.9804	1	0.9804	0.1817	0.6918	-
AB^2^	8.45	1	8.45	1.57	0.2789	-
A^3^	0.0000	0	-	-	-	-
B^3^	0.0000	0	-	-	-	-
**Residual**	21.58	4	5.39	-	-	-
Lack of fit	15.28	1	15.28	7.28	0.0739	not significant
Pure error	6.30	3	2.10	-	-	-
**Cor total**	276.86	11	-	-	-	-

**Table 9:** Analysis of the variance of the regression model (after curing at 220 °C).

Source	Sum of Squares	df	Mean Square	F-Value	*p*-Value	Note
**Model**	884.67	7	126.38	29.04	0.0028	significant
A-SCMs/Cement	200.00	1	200.00	45.96	0.0025	-
B-Q/R	344.79	1	344.79	79.23	0.0009	-
AB	16.40	1	16.40	3.77	0.1242	-
A^2^	4.58	1	4.58	1.05	0.3631	-
B^2^	7.62	1	7.62	1.75	0.2564	-
A^2^B	35.23	1	35.23	8.09	0.0466	-
AB^2^	50.68	1	50.68	11.65	0.0270	-
A^3^	0.0000	0	-	-	-	-
B^3^	0.0000	0	-	-	-	-
**Residual**	17.41	4	4.35	-	-	-
Lack of fit	11.65	1	11.65	6.07	0.0906	not significant
Pure error	5.76	3	1.92	-	-	-
**Cor total**	902.08	11	-	-	-	-

**Table 10:** Thermal weight loss of cement samples cured at 180 °C.

Sample	Weight Loss (%)				
	0–340 °C	340–437 °C	437–574 °C	574–820 °C	820–1000 °C
HBC	15.33	13.97	29.78	27.68	9.20
HS40	58.57	14.13	13.27	11.39	4.64
HS40-9/1	55.33	14.66	13.57	9.35	4.00
HS40-4/1	51.94	15.50	15.71	11.48	3.38
HS40-7/3	48.97	17.74	16.30	12.38	4.60
HS40-3/2	46.12	18.48	17.61	14.67	5.12

## Data Availability

The data presented in this study are available upon request from the corresponding author.
